# circ0101675 promotes malignant process via sponging miR-1278 and upregulating WNT3A/5A in non-small cell lung cancer

**DOI:** 10.7150/jca.57255

**Published:** 2021-05-17

**Authors:** Wei Du, Jianpeng Hu, Rong Hu, Min Yang, Yun Peng, Zhiwei Zhang, Yuehua Li, Xiusheng He

**Affiliations:** 1 Key Laboratory of Cancer Cellular and Molecular Pathology in Hunan Province, Cancer Research Institute, University of South China, Hengyang 421001, Hunan Province, China.; 2 Department of pathology, The First People's Hospital of Changde City, Hunan Province.; 3 Department of Medical Oncology, the First Affiliated Hospital, University of South China, Hengyang 421001, Hunan Province, China.

**Keywords:** circ0101675, circular RNAs, WNT3A, WNT5A, competitive endogenous RNAs, non-small cell lung cancer

## Abstract

Circular RNAs (circRNAs) is one type of non-coding RNAs (ncRNAs) which have many roles in biological processes, as well as modulation intracellular gene expression modulation. Nonethless, the roles along with expression status of the most circRNAs in NSCLC (non-small cell lung cancer) remain unknown. Herein, we conducted a high-throughput microarray sequencing to identify abnormal expressed circRNAs. Circ0101675 was found upregulated in NSCLC cell lines and tissues. We carried out colony formation, transwell, CCK-8, and animal assays to investigate the functions of circ0101675. Silence of circ0101675 inhibited the migration and proliferation of NSCLC.

To elucidate the mechanism, RNA immunoprecipitation assays along with luciferase enzyme reporter assays were further employed to explore the cross-talk between circ0101675 and other molecules. We discovered that circ0101675 facilitates the malignant process of growth and migration via sponging miR-1278 and upregulating WNT3A/5A expression. In conclusion, we revelaed the vital role of circ0101675-miR-1278-WNT3A/5A signaling in NSCLC progression via the competing endogenous RNAs mechanism. Therefore, circ0101675 can be used as a new and useful biomarker for monitoring and treating NSCLC.

## Introduction

Based on the global cancer statistics, lung cancer is the most pervasive malignancy (over one in ten of the overall cases) and is the most frequent cause of cancer-linked deaths (about one in five of total cancer deaths) globally 1. Non-small cell lung cancer (NSCLC) is the most frequent subtype of lung cancer responsible for about 85% cases 2. Only about 15% patients diagnosed with NSCLC can be alive for more than 5 years 3. Once recurrence and metastasis occur, the outcome and the quality of life of NSCLC patients will become worse. Thus, to develop an effective therapeutic approach of NSCLC progression is of great importance.

Circular RNAs (circRNAs) is a new kind of single-lined non-coding RNAs transcripts, which regulates the expression of numerous key genes via interacting with microRNA (miRNA) or other molecules in different mechanism 4. circRNAs are endogenous noncoding RNAs ordinated from the back-spliced sequences of introns or exons of pre-mRNAs (precursor mRNAs) without a 5' head or a 3' tail which are abundant in most mammalian tissues 5. Compared to the linear mRNA counterparts, circRNAs are more stable in cells because of their peculiar circular structure which can resist to RNA exonuclease 6. As vital regulators of various biological processes in the cell, circRNAs change the expression of vital genes via multiple comprehensive molecular mechanisms, including binding microRNAs, binding proteins and encoding novel polypeptides 7. In the last decade, due to the development of RNA-sequencing technology and bioinformatic analysis, scientists are increasingly interested in the role of circRNAs during cancer process 8. For instance, the most famous circRNA ciRS-7/cdr1as increases the proliferation, invasion, drug resistance, and immune escape by sponging miR-7 in multiple types of cancers 9-13. Additionally, circRAD18 and circGNB1 were identified as oncogenes though the mechanism of ceRNAs in breast cancer 14,15. CircFBXW7 is absent in tumor tissues which can suppress cell multiplication and migration in glioma and triple-negative breast cancer by translating a FBXW7-185aa new protein and sponging miRNA 16,17. Originating from the second exon of the HIPK3 gene, circHIPK3 increases cell growth and metastasis by blocking multiple miRNAs (e.g. miR-124) 18. However, the underlying molecular mechanism and the potential roles of most circRNAs remain unclear in NSCLC, hence should be explored.

Herein, a novel circRNA (hsa_circ_0101675) was identified in NSCLC by high-throughput microarray. Upregulation of circ0101675 was found in NSCLC cells and tissues. Silencing of circ0101675 dramatically repressed the migration and growth ability in NSCLC cells and mouse xenograft models. We next investigate the molecular mechanism of circ0101675 in NSCLC using conducted relevant experiments. All in all, our study uncovered the biological role of the circ0101675-miR-1278-WNT3A/WNT5A axis in NSCLC malignant process.

## Materials and Methods

### Clinical sample data

Fresh primary NSCLC tissues and neighboring non-malignant lung tissues were acquired from the First People's Hospital of Changde City and were frozen in liquid nitrogen immediately. This study was approved by the Ethics Committee of the First People's Hospital of Changde City and performed in accordance with the Declaration of Helsinki. All the study subjects granted written informed consent prior to participation in this study. The animal protocols were was approved and performed according to the guidelines of Institutional Animal Care and Use Committee of the First People's Hospital of Changde City.

### Microarray analysis

Four NSCLC tissues and matched neighboring non-malignant lung tissues from patients were assessed using Arraystar Human circRNA Array V2. RNase R was used to degrade linear RNAs to enrich circular RNAs. The labeled circRNAs were hybridized onto the Arraystar Human circRNA Array V2 (8x15K, Arraystar). Quantile normalization and consecutive data processing was carried using the R software (limma package).

### Cell culture

All cell lines including PC9, H1299, H1975, A549, and Beas2b used in this study were obtained from the ATCC. Cancer derived cell lines (PC9, H1299, H1975, and A549) were grown in DMEM (Gibco, USA) enriched with 10% FBS (Gibco). The normal human epithelial cell line Beas2b was cultured in BEBM media with additives (containing 0.01mg/ml fibronectin, 0.03 mg/ml bovine collagen type I as well as 0.01 mg/mL bovine serum albumin). The detection for mycoplasma infection was performed on a regular basis. Before experiment, the authenticity of all cell lines were used to verify the authenticity of all cell lines.

### RT-qPCR analysis

TRIzol (Invitrogen, USA) was utilized to extract total cellular RNA. qRT-PCR assays were carried out with SYBR Premix Ex Taq (Takara, Japan). The primers for circ0101675 are F: 5'-GAAGGCCCTCCACCTAACAC-3', R: 5'- AGCTGTCCAAAGTATGCTCAGT-3'. The primers for NPAS3 are F: 5'- TGTCTTTGACTATGTCCACCCC-3', R: 5'- GGGCTGGTTGACTCCACTG-3'. The primers for GAPDH are F: 5'-GGAGCGAGATCCCTCCAAAAT-3', R: 5'- GGCTGTTGTCATACTTCTCATGG-3'.

### Western blot analysis

The total protein from cells was isolated with RIPA lysis and added with PMSF. The protein was electro-blotted onto PVDF membranes for 2 hours at 300 mA. The membrane was inoculated with primary antibody (1:1000) and incubated overnight at 4 °C. After that, the membrane was inoculated with the secondary antibody at RT (room temperature) for 1 hour. Primary antibody anti-WNT3A (1:1000, Abcam, USA) and anti-WNT5A antibody (1:1000, Abcam, USA) are used to detect certain protein. The protein bands were exposed.

### Actinomycin D assay

H1299 and A549 cells were inoculated with 3 ug/ml actinomycin D (MCE) to degrade the linear mRNA transcription for 0, 8, 16, and 24 hours. Afterwards, we harvested the cells were harvested at certain time period and the linear NPAS3 mRNA and circRNA circ0101675 were tested by RT-qPCR analysis.

### RNase R digestion assay

After 2 ug extracted total RNA of H1299 and A549 cell line was incubated with RNase R (5 U/ug) or ddH2O for 30 minutes at 37 °C, the remaining RNA solution was purified and quantified by RT-qPCR analysis.

### Cell counting kit-8 assay (CCK-8)

The cells were resuspended, and si-circ0101675 (5000 cells per well) and si-circCTR cancer cells (5000 cells per well) were planted into a 96-well plate. The cells were incubated for 1, 2, 3, as well as 4 days at 37 °C. After that, CCK-8 solution (10 μl) was added to each small well of the plate before incubating for 2 hours. Using a microplate reader, the absorbance of each well was measured at 450 nm.

### Colony formation assay

A total of 5×10^3^ H1299 and A549 NSCLC cells were plated and incubated in each well of a 6-well plate. After incubation at 37 °C for 14 days. Thereafter, colonies were fixed with methanol and stained with 0.1% crystal violet. Subsequently, ImageJ software was employed to determine the colony number.

### Transwell assay

Overall, 3×10^4^ cells were resuspended and introduced to the upper compartments (medium without FBS) and medium (medium containing 20% FBS) was added to the lower compartments. Then, cells in the upper compartments were discarded. After fixing and staining with crystal violet (1%), the migrated cells were imaged and counted under a microscope (Nikon Instruments, NY, USA).

### Luciferase reporter assay

H1299 and A549 cells were seeded into 3 × 10^4^ cells per well (96-well plate). The predicted miR-1287 docking sites of circ0101675, 3'-UTR of WNT3A, and 3'-UTR of WNT5A was mutated. The miRNA inhibitors or mimics and constructed reporting vectors (circ0101675-wt/mut or WNT3/5A 3'-UTR-wt/mut) were co-inserted into cells for 48 hours via co-transfection. Dual-luciferase reporter assay system kit (Promega) was employed to evaluate relative luciferase enzyme activity.

### RNA immunoprecipitation (RIP)

H1299 and A549 cells were inserted with MS2bs-circ0101675, MS2bs-circ0101675-mt and MS2bs-Rluc via transfection. After incubating for 48 hours, RIP was performed. The level of miR-1278 was determined after the purification of RNA complexes. The RIP assays for AGO2 protein were performed with an anti-Ago2 antibody. The relative abundance of circ0101675, WNT3A, WNT5A and miR-1278 was tested after RNA purification.

### Mouse xenograft model

A549 cells (1×10^7^) were subcutaneously administered into nude mice (five mice per group, 5-week-old) and administered with intratumoral injection (50 μL si-circCTR, or si-circ0101675) every four days. The volume of tumors was estimated every four days according to the following formula: 0.5×width^2^×length. After four weeks, we euthanized the mice were administered and the tumors were weighed. Cells (2 × 10^5^) were injected through tail veins of nude mice (six mice per group) via mouse lung metastasis assay. The lungs were extracted after eight weeks and the number of metastatic sites were quantified via microscopy of HE-stained sections.

### Statistical analysis

All statistical analysis was performed with SPSS 22.0 software (SPSS Inc., Chicago, IL, USA). All data are reported as the mean ± standard deviation (SD). The expression of circ0101675 in two matched groups was compared using Paired t test. Groups were compared using Student's t test. *P*<0.05 signified statistical significance.

## Results

### Circ0101675 is upregulated in NSCLC

**Figure 1:**
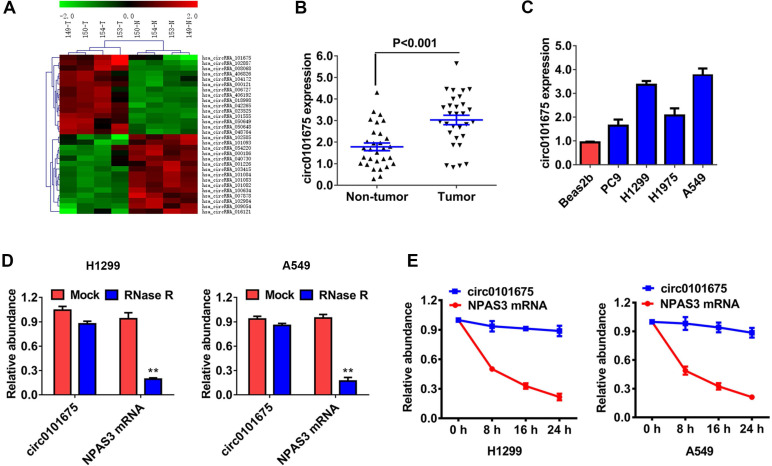
** Circ0101675 is upregulated in NSCLC with circular characteristics. (A)** Cluster heat map illustrating circRNAs which are differentially expressed. **(B)** The relative expression level of circ0101675 between NSCLC tissues and neighboring non-malignant lung tissues. **(C)** The relative expression level of circ0101675 in non-malignant lung cell line and cancer cell lines. **(D)** RNase R assay examined the circular structure of circ0101675 in H1299 and A549 cell line. **(E)** Circular transcripts of circ0101675 were more stable than its linear mRNA transcripts determined by actinomycin D treated assay in H1299 and A549 cell line.

To explore the possible involvement of circRNAs, a high-throughput circRNAs microarray assay was performed using four pairs of NSCLC patient tissues (Figure 1A). We found that circ0101675 was the most up-regulated circRNA between non-small cell lung cancerous tissues and non-cancerous matched non-malignant lung tissue. Afterwards, we assessed the expression level of circ0101675 in thirty paired cancer tissues and the neighboring non-malignant lung tissues by RT-qPCR analysis. We established that circ0101675 expression in NSCLC tissues was remarkably upregulated (Figure 1B). Consistently, circ0101675 was overexpressed in cancer cell lines in contrast with non-malignant lung cell line, particularly in H1299 and A549 cell lines (Figure 1C). RNase R assays and actinomycin D assays were then carried out to explore the circular structure and stability of circ0101675. The results showed that circ0101675 was resisted to RNA exonuclease with longer half-life span than the linear mRNA in H1299 and A549 cell lines (Figure 1D-E).

### Knockdown of circ0101675 decreases the growth and metastasis of NSCLC cells *in vitro*

**Figure 2:**
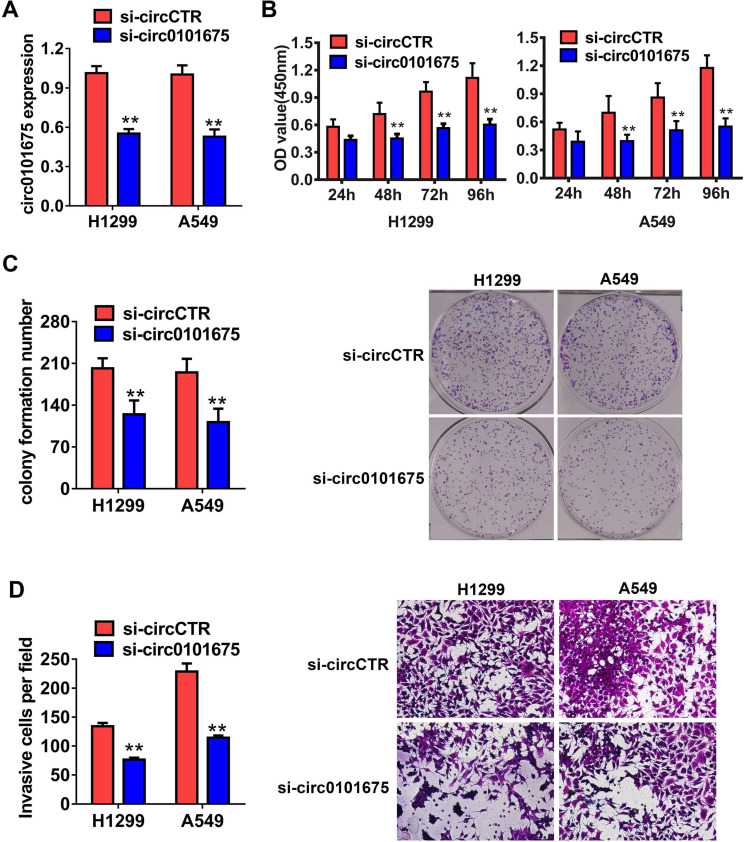
** Knockdown of circ0101675 decreases the growth and metastasis of NSCLC cells *in vitro*. (A)** Knockdown efficacy of circ0101675 was validated in H1299 and A549 cell line, assessing by qRT-PCR analysis. **(B)** After knockdown of circ0101675, CCK-8 assays were carried out. **(C)** Colony formation assays revealed that circ0101675 silencing suppressed cell colony formatting ability. **(D)** Transwell assays evaluating cell migration capability in H1299 and A549 cell line. ^*^P<0.05; ^**^P<0.01.

Then, functional assays were employed to assess the possible role of circ0101675 in tumor progression. The expression level of circ0101675 was diminished after transfection with siRNA (targeting the back-splicing junction region of circRNA) which verified the efficacy of the knockdown assay in H1299 and A549 cell lines (Figure 2A). CCK-8 assays showed that downregulation of circ0101675 suppressed multiplication ability of H1299 and A549 cell lines *in vitro* (Figure 2B). circ0101675 downregulation inhibited cell colony formatting ability of cancer cells, validated by colony formation assays (Figure 2C). Transwell assays revealed that circ0101675 silencing suppressed infiltration potential of cancer cells (Figure 2D).

### Knockdown of circ0101675 decreases the NSCLC cell growth and metastasis of non-small cell lung cancer cells *in vivo*

We next conducted animal assays to further validate the role of circ0101675 in mouse xenograft model. Tumor volumes measured at each time point showed that suppression of circ0101675 could remarkably inhibit tumor growth (Figure 2A-B). In addition, Ki67 protein levels in tumor mouse xenografts of two groups were analyzed by immunohistochemistry. In the circ0101675 knockdown group, the Ki67 expression was significantly reduced in tumor tissues (Figure 2C). Lung metastasis assays showed that downregulation of circ0101675 could reduce the ability of tumors to colonize the lungs (Figure 2D-F).

### Circ0101675 sponges miR-1278 in NSCLC

**Figure 4:**
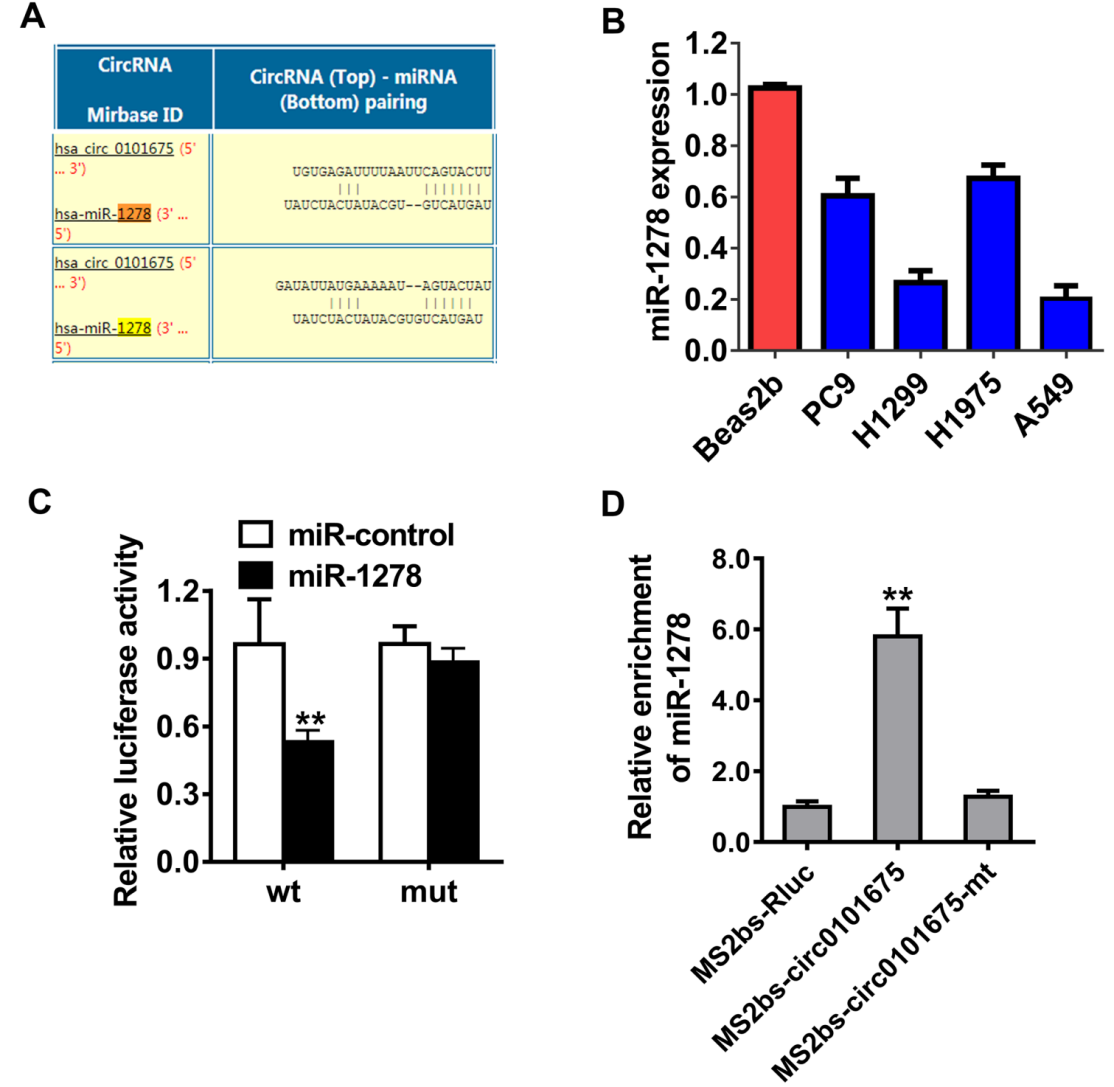
** Circ0101675 sponges miR-1278 in NSCLC. (A)** Predicted docking sites of miR-1278 within the circ0101675 sequence. **(B)** The relative expression level of miR-1278 in non-small cell lung cancer cell lines. **(C)** Luciferase reporter assay of H1299 cells inserted with miR-1278 mimics and circ0101675 wild type/mutant luciferase reporter via transfection. **(D)** MS2-based RIP assay transfected with MS2bs-circ0101675, MS2bs-circ0101675-mt or Rluc control. ^*^P<0.05; ^**^P<0.01.

In order to explore the mechanism by which circ0101675 promotes cancer progression, Circular RNA Interactome database was used to evaluate the potential interaction between circRNA and miRNAs. Among all the candidates, miR-1278 was predicted to interact with circ0101675 (Figure 4A). In NSCLC cell lines, miR-1278 was downregulated detected by RT-qPCR as indicated in Figure 4B. Dual luciferase reporter assays illustrated that the relative luciferase enzyme activity was remarkably diminished after the co-transfection of miR-1278 mimics, as well as wild type vectors (Figure 4C). Then, we conducted Ago2-related RIP assays to validate the direct cross talk of circ0101675 with miR-1278. The data illustrated that miR-1278 was predominantly gathered in the MS2bs-circ0101675 group (Figure 4D).

### Circ0101675 enhances the progress of NSCLC via the circ0101675-miR-1278-WNT3A/WNT5A cascade

**Figure 5:**
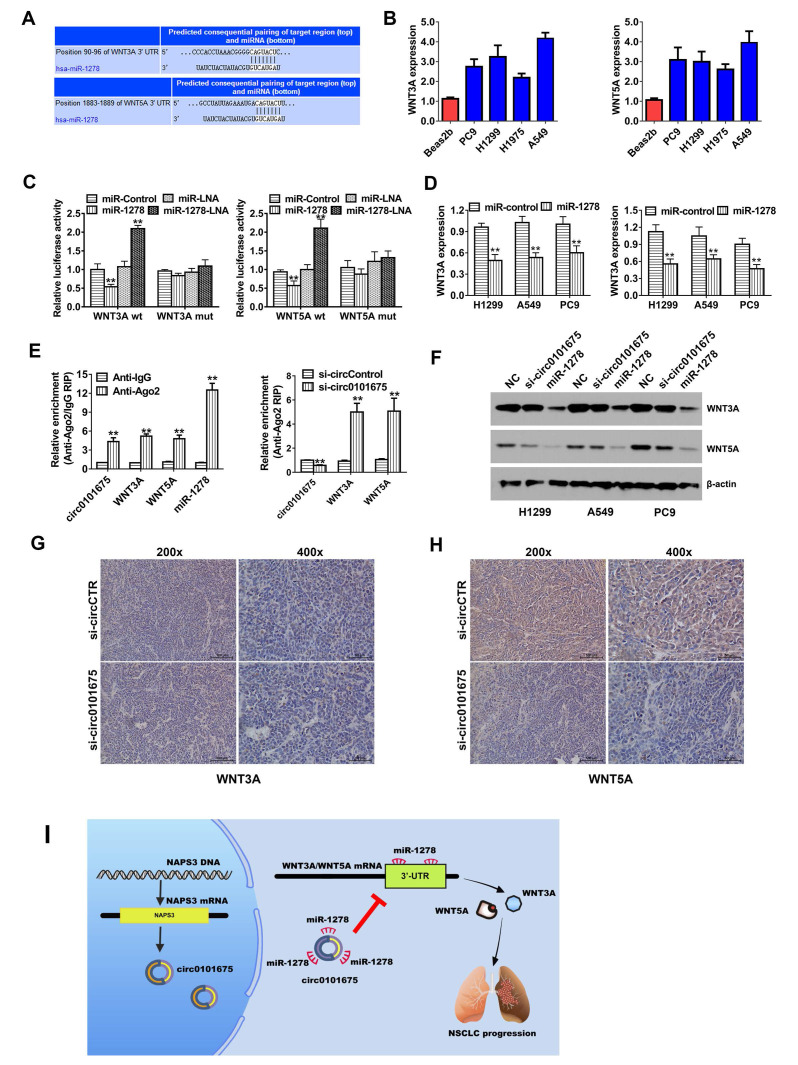
** Circ0101675 enhances the progress pf NSCLC via circ0101675-miR-1278-WNT3A/WNT5A cascade. (A)** Predicted interacting sites of miR-1278 within the 3'-UTR of WNT3A/5A mRNA by the TargetScan online website. **(B)** WNT3A/5A expression in NSCLC cell lines. **(C)** Luciferase enzyme reporter assay of H1299 and A549 cells co-inserted with miR-1278 mimics and the 3'-UTR of WNT3A/5A wild type/mutant luciferase reporter via co-transfection. **(D)** Detected by qPCR analysis, expression of WNT3A/5A was reduced after transfection with miR-1278 mimics. **(E)** Enrichment of circ0101675, WNT3A/5A and miR-1278 on AGO2 RNA binding protein. Enrichment of WNT3A/5A to AGO2 was increased after knockdown of circ0101675. **(F)** Silencing of circ0101675 or overexpression of miR-1278 decreased WNT3A/5A expression detected by western blot analysis. **(G)** The immunohistochemistry analysis was performed for xenograft tumors and the representative images of WNT3A expression are presented. **(H)** Immunohistochemistry images illustrating WNT5A expression in xenograft tumors. **(I)** A schematic model showing that circ0101675 promotes malignant process via sponging miR-1278 and upregulating WNT3A/5A in NSCLC. ^*^P<0.05; ^**^P<0.01.

TargetScan algorithm was employed to forecast the downstream genes of miR-1278. Among the genes, WNT3A and WNT5A were identified as downstream target oncogenes (Figure 5A). WNT3A and WNT5A were proteins have been implicated in several biological processes, including regulation of cell fate and cancer proliferation. Detected by RT-qPCR analysis, WNT3A and WNT5A were found upregulated (Figure 5B). Luciferase enzyme activity was remarkably diminished after the transfection of miR-1278 mimics and WT 3'-UTR- WNT3A and WNT5A reporter in H1299 and A549 cell lines. However, after transfection of the mutated reporting vector, this phenomenon was not further observed (Figure 5C). After transfection of miR-1278 mimics, the transcription expression level of WNT3A and WNT5A was reduced, indicating that WNT3A and WNT5A are downregulated by miR-1278 (Figure 5D). Furthermore, AGO2 related RIP assays showed that circ0101675, miR-1278 and WNT3A/5A were all enriched to AGO2 RNA binding protein in both H1299 and A549 cancer cells (Figure 5E). Knockdown of circ0101675 could remarkably increase WNT3A/5A enrichment to RNA induced silencing complex (RISC) (Figure 5E). Analyzed by western blot assays, overexpression of miR-1278 and silence of circ0101675 could incredibly decrease the protein level of WNT3A and WNT5A (Figure 5F). Additionally, WNT3A and WNT5A protein levels in tumor mouce xenografts of two groups were analyzed by immunohistochemistry. In the circ0101675 knockdown group, the WNT3A and WNT5A expression was significantly reduced in tumor tissues (Figure 5G-H).

## Discussion

Unlike the linear structure of mature mRNA with start and stop termini, circRNAs are a class of novel non-coding RNAs which extensively expressed in mammal tissues with covalently closed loop 5. These kind of special non-coding circular RNAs are not useless products of RNA splicing but are very crucial regulators in cells 19. In recent years, thousands of circRNAs were discovered as novel monitoring biomarkers and promising treatment targets for tumor therapies 8. More and more circRNAs have been verified and well-examined in the field of tumour research in different kinds of cancer. A circRNA derived from CTNNB1 locus promotes hepatocellular carcinoma cell progression via encoding a new 370-aa β-catenin protein which stimulating the Wnt signaling pathway 20. circPLK1 were identified as a tumor promoting circRNA by reducing apoptosis in breast cancer 21,22. Circular RNA circRIMS1 promotes malignant process by binding miR-433-3p and upregulating CCAR1 expression in bladder cancer 23. In TNBC, CircRNA derived from AHNAK1 reduces tumor progression by interacting with RASA1 and miR-421 24. However, there are only few researches investigating the functions roles and of circRNAs in NSCLC. For instance, circPRKCI is upregulated in lung cancer which promotes tumorigenesis via sponging miR-589 and miR-545 25.

In the current study, circ0101675 was found upregulated in tissues along with the cell lines by a high-throughput microarray sequencing in NSCLC. Knockdown of circ0101675 could remarkably repress NSCLC cell growth and migration. Besides, relevant experiments and assays were further conducted to explore the molecular mechanism of circ0101675. The outcomes suggested that circ0101675 promotes the malignant progression of NSCLC by sponging miR-1278 and upregulating WNT3A and WNT5A expression.

According to the published studies, miR-1278 was discovered to inhibit cancer progression in many kinds of cancers. For instance, miR-1278 is downregulated and inhibits glioma cell proliferation by degradation of neurofilament medium (NEFM) 27. Similarly, miR-1278 could also be sponged by long non-coding RNA LINC00294 in glioma cell. Though targeting ATG2B, miR-1278 suppresses autophagy and sensitizes cells to cisplatin in nasopharyngeal carcinoma 28. In clear cell renal cell carcinoma, miR-1278 was differentially expressed which regulated by three circRNAs and could provide some potential therapeutic options for clear cell renal cell carcinoma treatment 29. As a target downstream of miR-1278, WNT3A and WNT5A are important regulator of canonical Wnt signaling pathway 30. WNT3A regulates cancer cell stemness and increases metastatic potential via CTNNB1 signaling pathway and upregulation of Notch3 31,32. Another Wnt family member, WNT5A enhances brain metastasis of through the ERK1/2 cascade in EGFR-mutant NSCLC 33. In our research studying NSCLC, WNT3A and WNT5A were validated to be the target of miR-1278 in NSCLC. Circ0101675 could increase the expression of WNT3A and WNT5A by the ceRNA mechanism.

In summary, our study demonstrated the core role of circ0101675 in NSCLC progression via the mechanism of ceRNA. Thus, circ0101675 might act as a new and useful marker for monitoring and treatment target for non-small cell lung cancer.

## Figures and Tables

**Figure 3:**
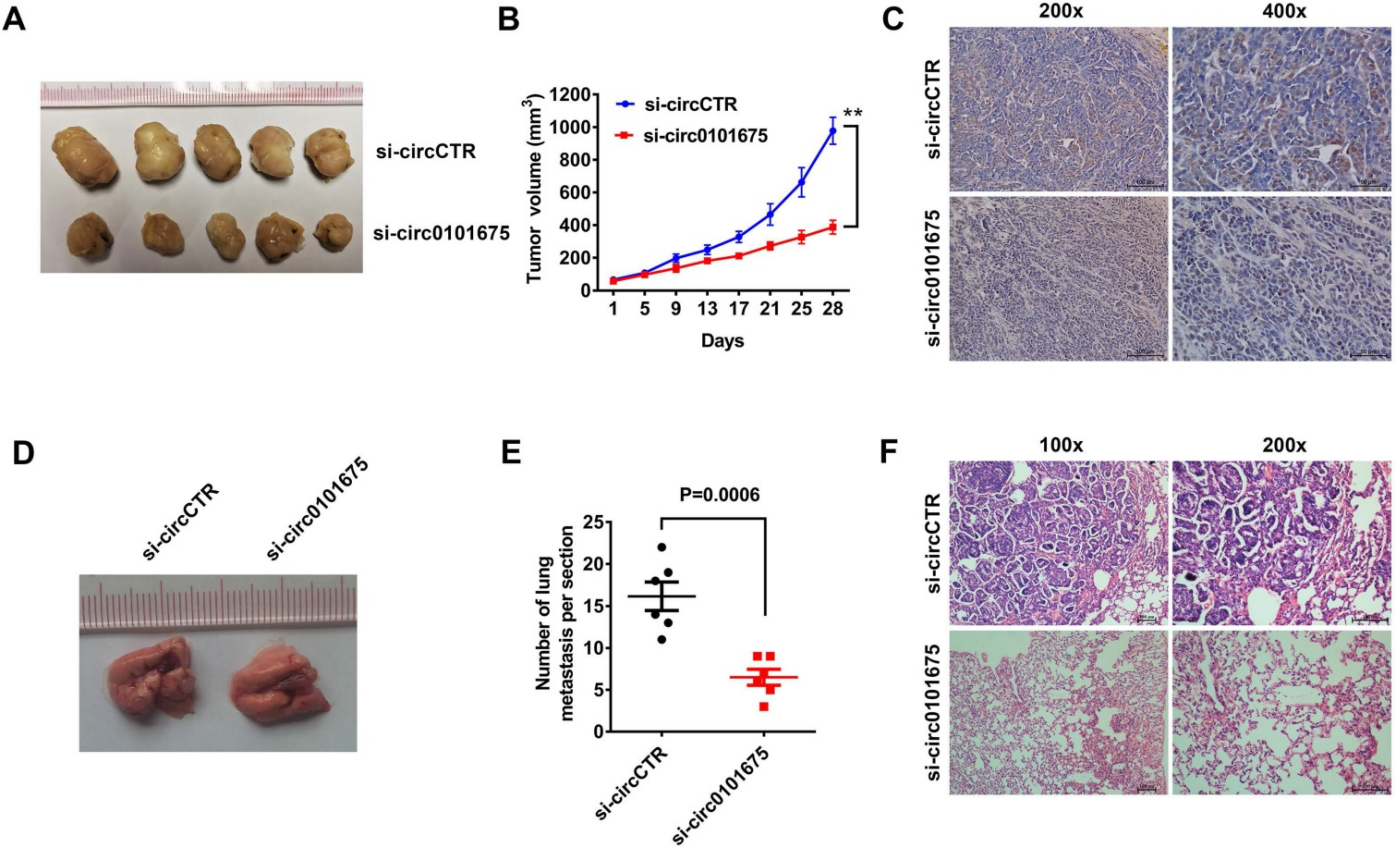
** Silencing of circ0101675 decreases the growth and metastasis of NSCLC cells *in vivo*. (A)** Mouse xenograft models of A549 cell line were established. **(B)** Tumor volume was estimated in every four days. **(C)** Images illustrating immunohistochemistry assessment of Ki-67 expression in xenograft tumors. **(D-F)** The total number of lung metastases was counted and recorded. HE-stained sections of lung metastases were presented.
